# Gut microbiota plays a pivotal role in opioid-induced adverse effects in gastrointestinal system

**DOI:** 10.1186/s13054-021-03867-0

**Published:** 2022-01-03

**Authors:** Rongpeng Xu, Liying Miao, Chun Yang, Bin Zhu

**Affiliations:** 1 Department of Critical Care Medicine, The Third Affiliated Hospital of Soochow University, Changzhou, 213000 China; 2 Department of Nephrology, The Third Affiliated Hospital of Soochow University, Changzhou, 213000 China; 3 Department of Anesthesiology and Perioperative Medicine, The First Affiliated Hospital of Nanjing Medical University, Nanjing, 210029 China

**Dear Editor,**

Recently, we read with great interest the article by Yan et al. [1], in which the authors reviewed the effect of opioids on gastrointestinal function in ICU patients. This review deepens our understanding of the opioid analgesia in ICU patients; however, the mechanisms of gastrointestinal dysfunction induced by opioids need to be further elucidated.

As noted by the authors, 63–86% of ICU patients were treated with opioids, associated with a significantly higher incidence of gastrointestinal dysfunction [1]. More importantly, opioid-induced bowel dysfunction is associated with poor outcomes in critically ill patients. The authors suggest that opioids in the enteric nervous system inhibit neuronal excitability and cause neurotransmitter release imbalance via binding to opioid receptors, leading to gastrointestinal dysfunction [1]. This might partially explain that opioid antagonists are intended to treat opioid-induced constipation. However, current studies have found that the effects of opioid antagonists on gastrointestinal function are contradictory [1]. Meanwhile, the use of opioid antagonists can also cause serious adverse reactions, such as reversing the protective effect of opioids on inflammatory lung injury [2]. Thus, it is of great importance to further study the mechanism of gastrointestinal dysfunction caused by opioids and to find novel treatment targets.

It is well recognized that opioids could alter the composition and function of gut microbiota [1]. In addition, it should be noted that dysbiosis of gut microbiota can cause intestinal wall edema and abnormal production of microbial metabolites, resulting in gastrointestinal dysmotility and intestinal absorption dysfunction [3]. Interestingly, opioids can modulate 5-hydroxytryptamine (5-HT) metabolism, and dysfunctional 5-HT signaling may underlie the mechanisms of gastrointestinal disorders. Meanwhile, studies have shown that gut microbiota can regulate 5-HT synthesis [4]. Furthermore, opioids have obviously inhibitory effects on the central nervous system and can affect intestinal function through the gut brain axis, and that gut microbiota is an important part of the gut brain axis [5]. These suggest that the gut microbiota is critical in opioid-induced gastrointestinal dysfunction.

Given the extensive use of opioids for analgesia in ICU patients, it should be paid attention to explore the specific mechanism of gastrointestinal dysfunction caused by opioids. Dysbiosis of gut microbiota might play an important role in the mechanism of opioid-induced gastrointestinal adverse effects. Further detailed studies should focus on the effectiveness of probiotics, prebiotics and fecal microbiota transplantation in this important clinical issue.

## Data Availability

Not applicable.
