# Supplementary material for: Gut microbiota plays a pivotal role in opioid-induced adverse effects in gastrointestinal system
Source: Crit Care. 2022 Jan 3;26:5. doi: 10.1186/s13054-021-03867-0 (PMC8722173; doi:10.1186/s13054-021-03867-0)
Supplement: Related texts

# Reply: Authors’ response

**Authors:** Yun Yan, Yu Chen, Xijing Zhang

**Affiliations:** grid.417295.c 0000 0004 1799 374X Department of Critical Care Medicine, Xijing Hospital, The Fourth Military Medical University, Xi’an, China

We thank Xu et al. for their comments and interest in our review about the effect of opioids on gastrointestinal (GI) function in ICU patients [1]. Xu et al. expressed the effect of opioids on the alternation of the GI microbiota and the regulation of GI by the central nervous system through the gut microbiota.

Although we aimed to comprehensively and mainly focus on the direct effect of opioids in GI dysmotility in our review, we agree with the important role of gut microbiota on the GI and patients’ prognosis. And the benefit of gut microbiota on the GI function had been proved by probiotics, prebiotics and fecal microbiota transplantation [6]. However, the ICU-acquired gut microbiota dysbiosis included patient-related factors and modern ICU intervention [7]. The use of proton pump inhibitors, antibiotics, artificial nutrition and vasoactive agents may affect the gut microbiota [8]. Moreover, the previously reported significantly gut microbiota dysbiosis were stroke, transient ischemic attack, and chronic kidney disease [8]. Meanwhile, most of the articles regarding opioids’ effect on the microbiome was with morphine, and data about the impact of other types of opioids was little [6]. Additionally, the shift and the overgrowth of opioid-induced gut microbiota more resulted from the dysmotility of GI [6]. Whether the change of gut microbiota caused by opioids had an important role in mediating the GI function was uncertain. And the proportion of cause and consequence of microbiota dysbiosis on the GI injury in the ICU patients was unclear.

GI injury in ICU patients is multifactorial, and the management of critically ill patients with GI dysfunction is complex. We recognize that there are many mechanisms involved in the effect of opioids on the GI. We recognize the necessity of further studies that focus on the effect of opioids on the GI to provide more evidence on the clinical. Moreover, ICU patients are populations with complex conditions and higher heterogeneity. It should clarify whether the inhibition of opioids or the disturbance of opioid-induced gut microbiota on the GI function had a significantly important role in the ICU patients, and then optimize the drug administration for those who had a high risk of GI injury or already had GI injury.
